# Amoebal Tubulin Cleavage Late during Infection Is a Characteristic Feature of *Mimivirus* but Not of *Marseillevirus*

**DOI:** 10.1128/spectrum.02753-22

**Published:** 2022-12-01

**Authors:** Nisha Goyal, Amlan Barai, Shamik Sen, Kiran Kondabagil

**Affiliations:** a Department of Biosciences and Bioengineering, Indian Institute of Technology Bombay, Powai, Mumbai, India; Shandong First Medical University

**Keywords:** *Mimivirus*, *Marseillevirus*, *Acanthamoeba castellanii*, actin modification, tubulin cleavage

## Abstract

*Mimivirus* and *Marseillevirus* infections of Acanthamoeba castellanii, like most other viral infections, induce cytopathic effects (CPE). The details of how they bring about CPE and to what extent and how they modify the host cytoskeletal network are unclear. In this study, we compared the rearrangement of the host cytoskeletal network induced by *Mimivirus* and *Marseillevirus* upon infection. We show that while both *Mimivirus* and *Marseillevirus* infections of A. castellanii cells cause retraction of acanthopodia and depolymerization of the host actin filament network, the *Mimivirus* infection also results in characteristic cleavage of the host tubulin, a phenomenon not previously reported with any intracellular pathogens. Furthermore, we show that the amoebal tubulin cleavage during *Mimivirus* infection is a post-replicative event. Because time-lapse microscopy showed that *Mimivirus* infection leads to the bursting of cells, releasing the virus, we hypothesize that tubulin cleavage together with actin depolymerization during the later stages of *Mimivirus* assembly is essential for cell lysis due to apoptotic/necrotic cell death. We also characterize the *Mimivirus*-encoded gp560, a Zn metalloprotease, however, the purified gp560 protein was unable to cleave the commercially available porcine brain tubulin. While protein synthesis is essential for causing the morphological changes in the case of *Mimivirus*, the proteins which are packaged in the viral capsid along with the genome are sufficient to induce CPE in the case of *Marseillevirus*.

**IMPORTANCE** In general, intracellular pathogens target the cytoskeletal network to enable their life cycle inside the host. Pathogen-induced changes in the host cell morphology usually accompany global changes in the cytoskeleton resulting in cytopathic effects. While viruses have been shown to use the host actin cytoskeleton for entry and transport during early infection, the role of microtubules in the viral life cycle is only beginning to emerge. Here, we show that the giant viruses *Mimivirus* and *Marseillevirus* both induce depolymerization of the actin filament, *Mimivirus* also causes a characteristic cleavage of tubulin not previously reported for any intracellular pathogen. Because tubulin cleavage occurs late during infection, we hypothesize that tubulin cleavage aids in cell death and lysis rather than establishing infection. The different strategies used by viruses with similar host niches may help them survive in competition.

## INTRODUCTION

Virus infection of cells often induces cytopathic effects that are predominantly mediated by the reorganization of the host cytoskeleton (1–4). Cytoskeleton can act as both a barrier to virus infection and an abettor supporting the transport of incoming virus particles within the cell to the site of replication (5–8). Reorganization of the actin filaments which subsequently cause changes in cell shape and cell adhesion properties are believed to be the major causes of CPE. For example, during vaccinia virus (VV) infection, microtubule-dependent reorganization of actin filaments leads to the rounding of cells which is mediated by early viral proteins (1). Some nucleocytoplasmic large DNA viruses (NCLDVs) utilize host microtubules to form assembly sites known as viral factories (VF) early on and either disrupt microtubules or utilize them for egress at a later stage (9–11). The protozoan NCLDVs, such as *Mimivirus* (Acanthamoeba polyphaga *Mimivirus*) and *Marseillevirus* (A. polyphaga *Marseillevirus*), also induce CPE in the laboratory hosts A. polyphaga (AP) and A. castellanii (AC) which are characterized by rounding of cells, loss of motility, de-adhesion, and cell lysis (12–14).

Intracellular bacterial pathogens that infect and replicate within eukaryotic cells are also known to alter the host cytoskeleton network (15). Well-studied examples include human pathogens such as Chlamydia, *Rickettsia*, Salmonella, and *Shigella*. Free-living amoebae that are host to several NCLDVs are also infected by bacterial pathogens such as *Legionella. Rickettsia* induces actin polymerization to aid in its motility inside the cell (16). The RickA protein of Rickettsia conorii activates the Arp2/3 complex. A *Rickettsia* WASP-like protein activates the Arp2/3 complex and mediates actin-based motility (17). Chlamydia reorganizes the host cytoskeleton around host-membrane derived replication centers known as inclusions (18). Actin and intermediate filaments stabilize the Chlamydia trachomatis vacuole by forming dynamic structural scaffolds (19). Salmonella utilizes microtubule for the formation of replicative niches at the perinuclear site, *Shigella* disrupts microtubule structure to sustain intracellular actin-based bacterial motility to the periphery (20–24). *Legionella*, on the other hand, alters the actin cytoskeleton using different mechanisms (25–28).

*Mimivirus* and *Marseillevirus* are icosahedral, non-enveloped, large double-stranded DNA viruses which code for 979 and 457 proteins, respectively. This large repertoire of proteins enables a completely cytoplasmic life cycle in these viruses, but with significant interaction with the nucleus of the amoeba (14, 29–32). Omics studies have revealed that both *Mimivirus* and *Marseillevirus* particles package several proteins and mRNAs along with genome in virion particles which play essential roles during the early stages of infection (29–33). Within 1 h of entry, the fusion of *Mimivirus*-containing phagosome with lysosomes leads to the release of *Mimivirus* core into the cytoplasm, while *Marseillevirus* core is released through an opening of the vacuole membrane. It has been suggested that the transcription of some viral genes is initiated before the release of the *Mimivirus* core (34–36). Unlike in *Mimivirus*, the virus-encoded proteins related to transcription are not incorporated within the *Marseillevirus* virions. Thus, in the case of *Marseillevirus*, to initiate viral replication, it is reported that some nuclear proteins are transiently recruited to the VF (34). Between 2 and 4 h postinfection (pi) of *Marseillevirus*, diffusive VFs are formed near the nucleus. The *Marseillevirus* progenies begin to appear between 6 and 8 hpi. Both individual viral particles and several particles enclosed in large vesicles have been observed (29, 37). One clear distinction in the case of *Mimivirus* infection is that the individual replication centers (RCs), which are precursors to VF, coalesce with each other between 4 and 6 hpi to form a single large VF. From 8 hpi, fully assembled individual virus particles are continuously released into the cytoplasm from VF until cell lysis (38–41).

The changes in the cell morphology take place predominantly between 4 and 8 hpi during *Mimivirus* replication. This suggests that morphological changes predominantly occur after the VFs have been established. By the time new viral progenies start to accumulate in the cytoplasm, infected cells assume a spherical morphology and become characteristically immobile (41). Although *Marseillevirus* also induces rounding of *Acanthamoeba* during infection, they exhibit a different VF morphology. In addition, the replication cycle of *Marseillevirus* appears to be shorter than that observed for *Mimivirus* infection (41–43). In both *Mimivirus* and *Marseillevirus* infection, the causes of CPE leading to the rounding of cells are unclear.

In this study, we compared the morphological changes which occur in Acanthamoeba castellanii (AC) cells during *Mimivirus* and *Marseillevirus* infection. Most of the *Marseillevirus*-infected cells acquired rounded morphology early during infection, while *Mimivirus*-infected cells showed rounding at later time points, after the VF formation and presumably after the synthesis of early viral proteins. We also followed the changes in α-tubulin and polymerized and depolymerized forms of actin. We found that synthesis of *Mimivirus* early/late proteins is a prerequisite for the cleavage of host α-tubulin, while *Marseillevirus*-infected cells did not show tubulin cleavage. The cleavage of α-tubulin is a post-replicative event; cleaved tubulin was not detected before 6 h of *Mimivirus* infection. Additionally, since retraction of acanthopodia is observed in parallel with actin depolymerization during *Mimivirus* and *Marseillevirus* infection and the total amount of actin remained the same throughout the infection cycle, we presume that retraction of acanthopodia is a consequence of actin depolymerization.

## RESULTS

### CPE in *Mimivirus* and *Marseillevirus*-infected cells.

Although it has been reported that *Mimivirus* infection of *A. polyphaga* induces disruption of the host cytoskeletal components at around 4 hpi (41), the dynamics of the host cytoskeletal rearrangements induced by *Mimivirus* and *Marseillevirus* infections are poorly understood. We monitored changes in the arrangement of the host cytoskeleton upon infection of A. castellanii cells with *Mimivirus* and *Marseillevirus* using confocal microscopy. First, we optimized the protocol for the preservation of *Acanthamoeba* cytoskeleton during confocal microscopy. We found that a 3.5% paraformaldehyde in combination with 0.5% glutaraldehyde is sufficient to sustain the structure of cell cytoskeleton during confocal microscopy (Fig. S1A) and used this condition for all experiments.

**FIG 1:**
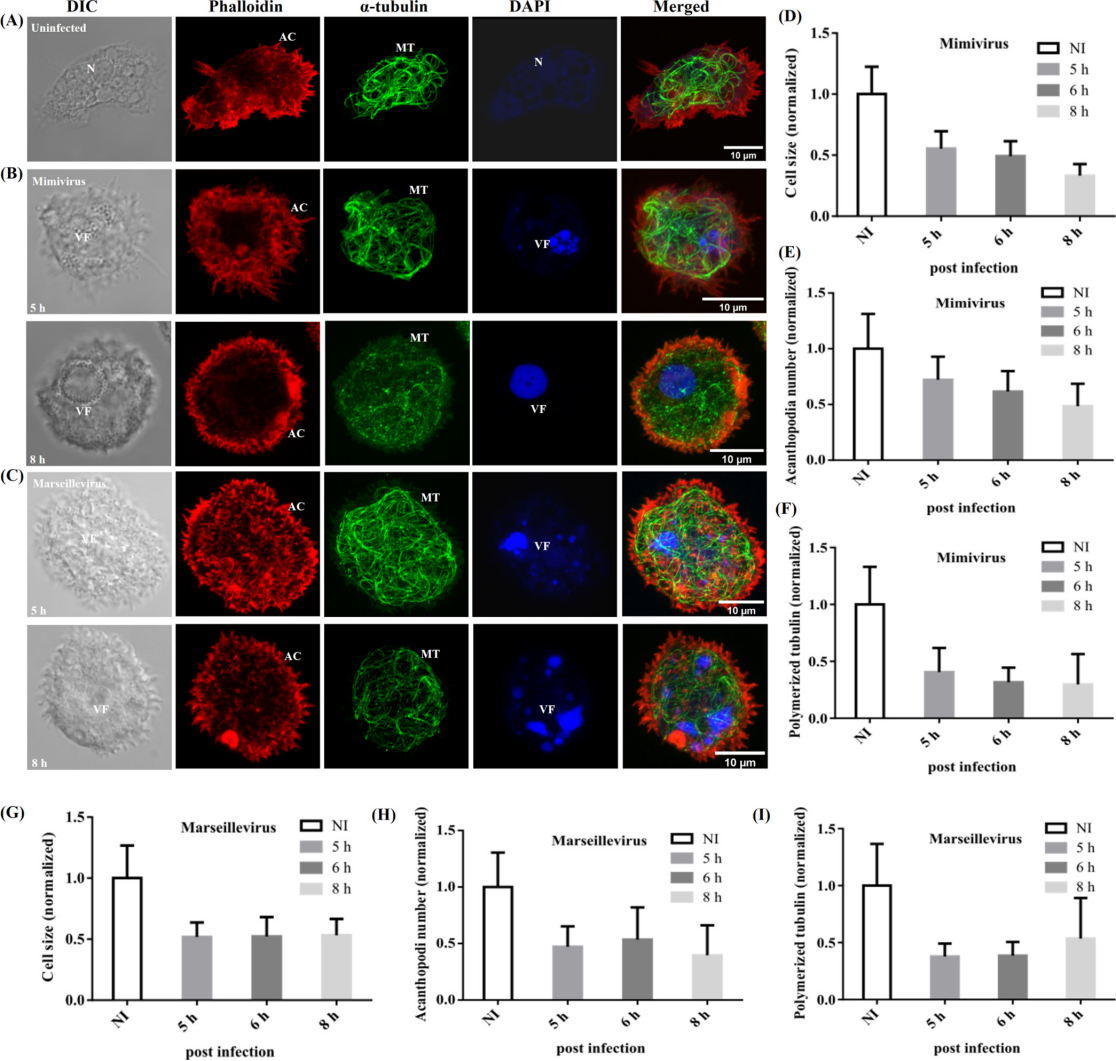
*Mimivirus* and *Marseillevirus* induced remodeling of cytoskeleton in Acanthamoeba castellanii. (A) Uninfected (B) *Mimivirus*-infected, and (C) *Marseillevirus*-infected *Acanthamoeba* were fixed at 5 and 8 hours post-infection (hpi) and stained with Alexa Fluor 555-labeled phalloidin (red) and yeast α-tubulin antibody (green), counterstained with DAPI (4′,6-diamidino-2-phenylindole, blue) and observed by confocal microscopy. The corresponding differential interference contrast (DIC) images were taken. Graphs showing quantification of cell area (D and G), number of acanthopodia (E and H), and polymerized tubulin (F and I) in *Mimivirus*- and *Marseillevirus*- infected cells using GraphPad Prism. Results are average from three independent experiments (*n* = 30 cells in each experiment) and error bars represent standard deviation (mean ± SD). VF, viral factory. N, nucleus. MT, microtubule. AC, acanthopodia. (Scale bar, 10 μm).

Compared to the uninfected A. castellanii cells (Fig. 1A), no significant changes in the arrangement of actin filaments and microtubules were observed until 4 hpi in *Mimivirus*-infected cells. Acanthopodia on the cell surface and microtubule loops within the subcortical region were intact at 4 hpi (Fig. S2A). In contrast, by 4 hpi, *Marseillevirus*-infected cells showed partial retraction of acanthopodia and a slight rearrangement of microtubules (Fig. S2B). *Mimivirus*-infected cells showed partial retraction of acanthopodia at around 5 hpi, but microtubule loops remained largely intact (Fig. 1B). By 5 hpi, *Mimivirus*-infected cells showed the presence of small discrete VFs while *Marseillevirus*-infected cells showed the presence of several small VFs in addition to a large VF (Fig. 1B and C). By 8 hpi, the *Mimivirus*-infected cells assumed a rounded morphology, and in most cases smaller VFs merged to form a single large VF (Fig. 1B and Fig. S2A). Interestingly, however, in a small fraction of the *Mimivirus*-infected cells, we observed the presence of two mature VFs, both producing progeny viruses (Fig. S2C and D). In the case of *Marseillevirus*-infected cells, by 5 hpi, cells were partially rounded, but smaller VFs formed and remained distinct throughout the infection cycle (Fig. 1C and Fig. S2B). Significant fragmentation and shortening of microtubules were observed in the *Mimivirus*-infected cells from 6 hpi onwards, which became more prominent between 8 and 12 hpi (Fig. 1B and Fig. S2A), while no such fragmentation of microtubules was evident in the *Marseillevirus*-infected cells (Fig. 1C and Fig. S2B). However, a comparable decrease in the loops of microtubules was observed in both *Marseillevirus*- and *Mimivirus*-infected cells.

Quantitative analysis further revealed that compared to the uninfected cells, at 8 hpi, *Mimivirus*- and *Marseillevirus*-infected cells showed about 67%, 52%, and 70% (Fig. 1D to F) and 48%, 61%, and 47% reductions in cell size, acanthopodia number, and polymerized tubulin, respectively (Fig. 1G to I). Furthermore, the decrease in cell size was compensated by an increase in cell height (Fig. S2E, compare panel i with panels ii and iii). Together, these results indicate that during both *Mimivirus* and *Marseillevirus* infections, CPEs become prominent only after the formation of VFs.

### Mimivirus infection induces depolymerization of actin and cleavage of tubulin.

To confirm whether the decrease in the number of acanthopodia and microtubule loops in infected cells resulted from depolymerization or a reduced amount of the total actin/tubulin, we performed a Western blot analysis with lysates collected from the infected cells at different infection time points.

**FIG 2:**
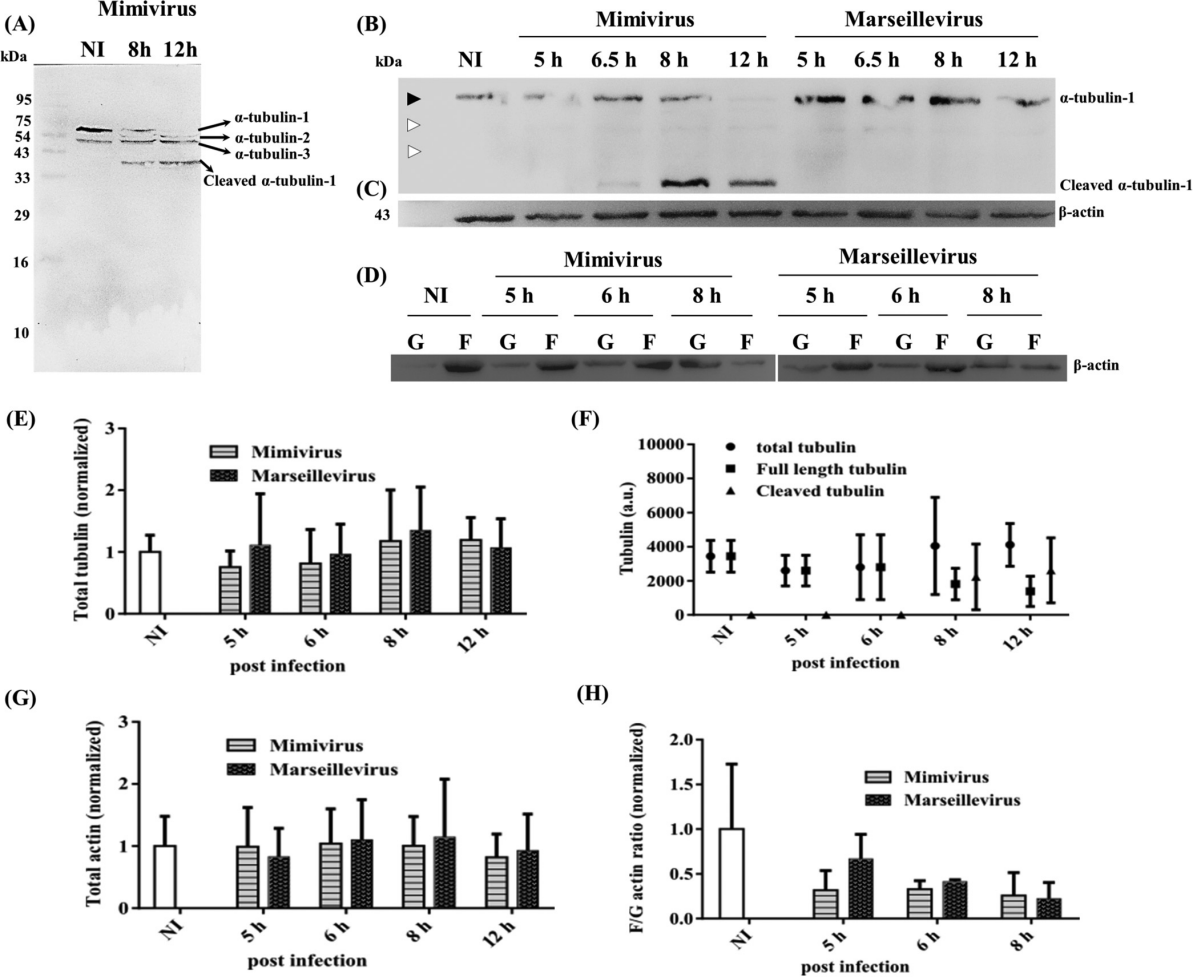
Western blot analysis and quantification of tubulin cleavage and actin depolymerization during *Mimivirus* and *Marseillevirus* infections of Acanthamoeba castellanii. Representative immunoblots: (A, B) showing progressive cleavage of α-tubulin during *Mimivirus* infection but not during *Marseillevirus* infection, (C) total actin, and (D) F- and G-actin fractions during *Mimivirus* and *Marseillevirus* infections. Equal amounts of protein were loaded in each lane. Three or more independent experiments were performed. (E to H) Quantitative analysis of total α-tubulin (E and F), total actin (G), and F/G actin ratio (H). Band intensity was measured using ImageJ software. Three forms of *Acanthamoeba* α-tubulin were seen. Upper cleaved fragment is indicated by black arrowhead; lower uncleaved fragments are indicated by white arrowheads. Error bars represent standard deviation (mean ± SD).

Previously, only a single form of the α-tubulin (~410 amino acids [aa], 45.3 kDa, GenBank accession no. DQ099493) was reported in A. castellanii (44). However, the UniproKB shows the presence of two additional forms of α-tubulin in A. castellanii of 460 aa (50.8 kDa, GenBank accession no. ELR12732) and 421 aa (46.5 kDa, GenBank accession no. ELR21488) in length, respectively. In the manuscript, we refer to the three forms of the α-tubulin as α-tubulin-1, -2, and -3 based on the molecular mass seen on the Western blot (α-tubulin-1 corresponds to 50.8 kDa). Although all three forms of α-tubulin could be detected by Western blotting when antibody specific to α-tubulin was used with A. castellanii lysate (Fig. 2A, lane 1), α-tubulin-1 appeared to be predominantly expressed (Fig. 2A). Furthermore, the Western blot analysis of the lysates of *Mimivirus*-infected *A castellanii* cells collected at 8 and 12 hpi clearly showed that only α-tubulin-1 undergoes the characteristic cleavage into a ~36-kDa fragment (Fig. 2A). The cleaved fragment (~16 kDa) was not detected, most likely due to further degradation of the cleaved fragment by cellular proteases, as has been speculated for actin (45). The human cytotoxic lymphocyte protease granzyme M (a serine protease), granzyme B (a serine protease), and caspase 6 (a cysteine-aspartic protease) have been reported to cleave α-tubulin at leucine and/or aspartate residue(s) (46–48). Considering the molecular weight of the major cleaved fragment (~36 kDa), we predict D321 as a possible protease cleavage site on the *Acanthamoeba* α-tubulin-1 (Fig. S2F).

Further, we found that the total amount of tubulin remained constant in the uninfected and *Marseillevirus*-infected cells until 12 hpi. Interestingly, unlike the uninfected and the *Marseillevirus*-infected cells, the *Mimivirus*-infected cells exhibited a characteristic cleaved band of α-tubulin of about ~36 kDa by about 6.5 hpi, and by 12 hpi, the amount of cleaved tubulin was increased (Fig. 2B).

No difference in the total amounts of actin between uninfected, *Mimivirus*-infected, and *Marseillevirus*-infected cells was observed (Fig. 2C). Next, to assess changes in the actin dynamics in *Mimivirus*- and *Marseillevirus*-infected cells, we followed a protocol previously used for the separation of soluble G-actin and insoluble F-actin (see Materials and Methods for details). Soluble G-actin in the cell lysate was separated from F-actin by low- and high-speed centrifugation steps, and the amount of actin present in the pellet and supernatant was analyzed by Western blotting. This analysis revealed that the fractions collected from the uninfected cells showed larger amounts of polymerized actin in the pellet compared to the supernatant fraction, while *Mimivirus*- and *Marseillevirus*-infected cells showed more actin in the supernatant fractions at 8 hpi (Fig. 2D).

Quantitative analysis of the total actin/tubulin at different infection time points also confirmed no significant changes in the total tubulin amount between *Mimivirus*- and *Marseillevirus*-infected cells until 8 hpi (Fig. 2E). Although the total amount of tubulin remained constant, with an increase in the cleaved tubulin, a corresponding decrease in the full-length tubulin band was observed from 6.5 hpi onwards (Fig. 2F). Although the total amount of actin remained constant throughout the infection (Fig. 2G), compared to that in uninfected cells, a significant decrease in the F/G ratio was observed from 8 hpi in both *Mimivirus*- and *Marseillevirus*-infected cells (Fig. 2H). Together, these data indicate that although both *Mimivirus* and *Marseillevirus* infections induce actin depolymerization, only *Mimivirus* infection results in the characteristic cleavage of α-tubulin.

### Comparison of lysis event and virus release dynamics in the *Mimivirus*- and *Marseillevirus*-infected cells.

**FIG 3:**
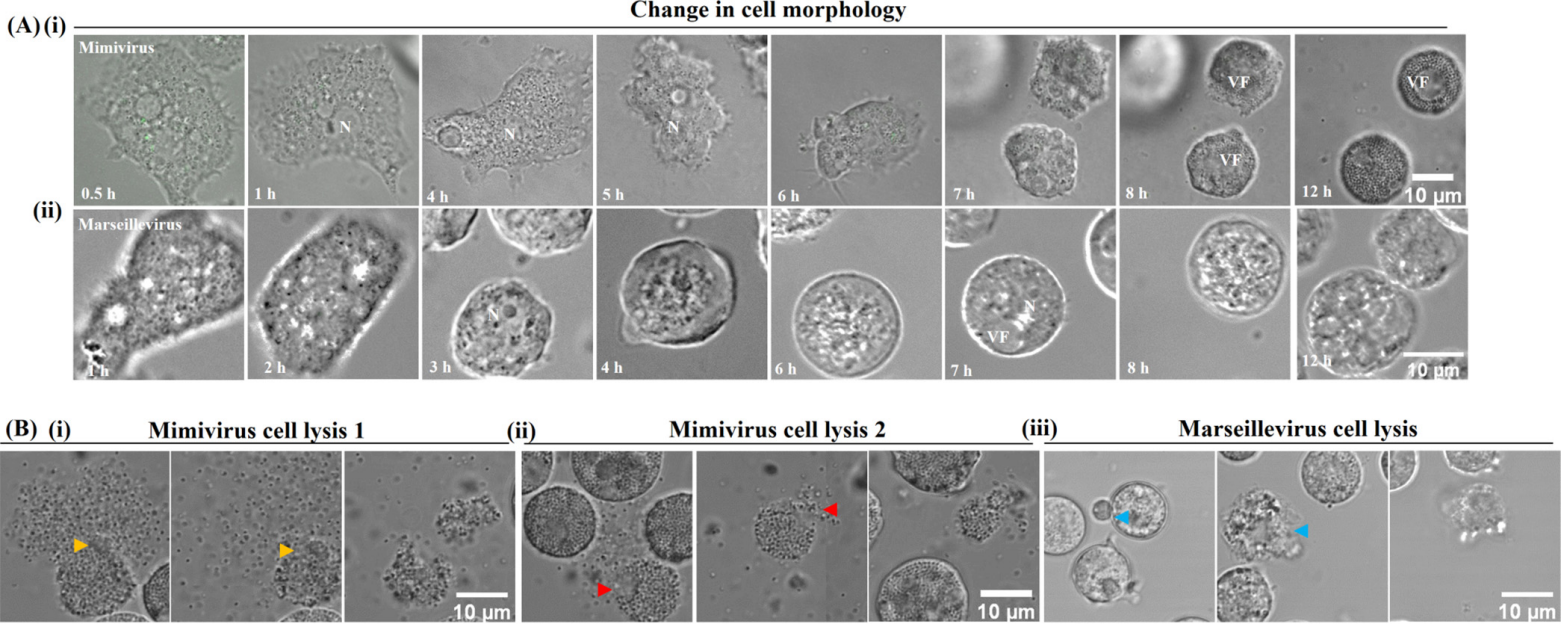
Time lapse microscopy of *Mimivirus*- and *Marseillevirus*-infected A. castellanii *cells*. (A) *Acanthamoeba* infected with SYBR Green I-stained *Mimivirus* (i) and *Marseillevirus* (ii). Representative images of *Mimivirus*- and *Marseillevirus*-infected cells at different times postinfection (pi) were extracted from Movies S1 to S5. Changes in the host cell morphology of infected cells were observed by DIC. (B) Snapshots of viral progeny release from *Mimivirus*- and *Marseillevirus*-infected cells. Most of the *Mimivirus*-infected cells underwent lysis due to bursting (i) (yellow arrowhead), while in some cells viral progenies were released slowly (ii) (red arrowhead). *Marseillevirus*-infected cells release viruses in the form of vesicles containing *Marseillevirus* (iii) (blue arrowhead). VF, viral factory; N, nucleus. Scale bar = 10 μm. Representative images of *Mimivirus*- and *Marseillevirus*-infected cells at different times pi were extracted from the Movies S1 to S5. To capture 30 to 40 cells in a single field, the “tile scanning” feature of the ZEN black software was used to stitch together multiple fields (see Materials and Methods for details). The vertical line in some images (and the accompanying movies) is a result of using the ‘tiling and stitching’ feature in Zen black software.

To further characterize the CPE, the changes in host cell morphology and host cell lysis were compared during the course of *Mimivirus* and *Marseillevirus* infections using time-lapse microscopy. AC cells were cultured in 1% low melting agarose containing the PYG medium (Fig. S1B). *Acanthamoeba* trophozoites were separately infected with SYBR Green I-stained virus particles and were time-lapse imaged from 0.5 to 24 hpi in the case of *Mimivirus*-infected cells and 2 to 44 hpi in the case of *Marseillevirus*-infected cells. There were no detectable changes in the morphology or motility of the amoebal cells infected with *Mimivirus* particles until 4 hpi. Reductions in cell size, motility, and morphological changes predominantly occurred between 5 and 6 hpi, and by 7 hpi, the *Mimivirus*-infected cells assumed a spherical shape (Fig. 3A, panel i; Movie S1). Similarly, most of the *Marseillevirus*-infected cells exhibited a reduction in the cell size at around 3 hpi and complete rounding by 4 hpi (Fig. 3A, panel ii; Movie S2).

By about 16 hpi, some of the *Mimivirus*-infected cells began to lyse. While many infected cells burst completely and released the progenies in a matter of seconds (Fig. 3B, panel i; Movie S3), some cells released the progeny viruses slowly over a period of 2 to 3 h where the bursting of cells was not apparent (Fig. 3B, panel ii; Movie S4). In contrast, there was no apparent bursting in *Marseillevirus*-infected cells. Instead, large vesicular enclosures containing several virus particles were released between 20 and 30 hpi (Fig. 3B, panel iii; Movie S5).

### VF establishment is necessary for inducing CPE during *Mimivirus* infection.

As shown in Fig. 1 and Fig. S2, both *Mimivirus*- and *Marseillevirus*-infected cells exhibited significant morphological changes only after VF formation. To test whether VF formation (and hence, presumably the synthesis of early viral proteins) in infected cells is essential for CPE, we infected amoebal cells with UV-inactivated virus particles. Our hypothesis here is that because UV treatment damages the viral DNA and stalls viral transcription and replication, it would help us understand whether the proteins packaged in the virus particles can trigger CPE or whether CPE requires the viral proteins synthesized in the VF upon infection. While *Mimivirus* particles treated with UV for up to 8 min exhibited normal CPE and replication, those with 12-min treatment failed to establish VF, and hence no morphological changes were observed in the infected cells even at a high multiplicity of infection (MOI) of about 100 (Fig. S3A to C). Similar observations were made with the heat-inactivated *Mimivirus* and *Marseillevirus* particles (Fig. S3D).

In the case of *Marseillevirus*, because the virions used for UV exposure were predominantly aggregated, a higher UV dosage was required to achieve complete inactivation. Thus, *Marseillevirus* particles exposed to UV for 30 min also failed to establish VF but, interestingly, could still induce CPE in the infected cells (Fig. S3A to C). In all cases, internalization of virus particles was not affected. When a higher MOI was used, a large number of internalized virus particles could be seen (Fig. S3D).

**FIG 4:**
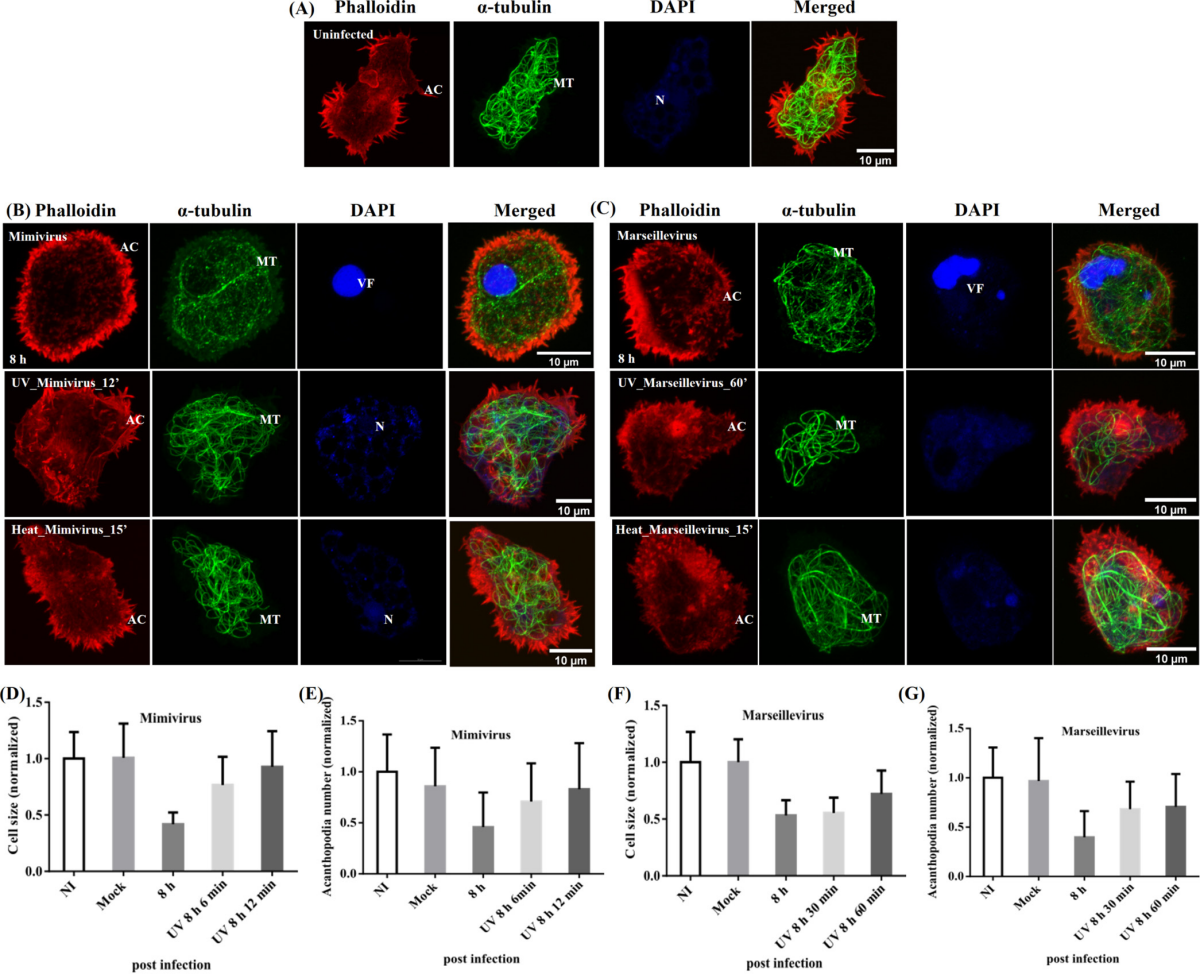
Requirement of virus protein synthesis in *Mimivirus*-induced cytopathic effects (CPEs) in A. castellanii cells. Cells were fixed and then stained with Alexa Fluor-555 labeled phalloidin (red) and yeast α-tubulin antibody (Green), counterstained with DAPI (blue), and analyzed using confocal microscopy at 8 hpi. (A) Uninfected *Acanthamoeba*. (B) *Acanthamoeba* infected with wild-type, UV-inactivated or heat-killed *Mimivirus*. (C) *Acanthamoeba* infected with wild-type, UV-inactivated, or heat-killed *Marseillevirus*. VF formation was not observed in either case. Graphs showing quantification of cell area and number of acanthopodia in *Mimivirus*- (D and E, respectively) and *Marseillevirus* (F and G, respectively)-infected cells using GraphPad Prism. Results are the average of three independent experiments (*n* = 30 cells in each experiment) and error bars represent standard deviation (mean ± SD). VF, viral factory. Scale bar = 10 μm.

In both cases of infection with UV and heat-inactivated *Mimivirus* particles, VF formation was not observed. There were also no appreciable changes in acanthopodia density and microtubule network morphology, comparable to that in the uninfected cells (Fig. 4A and B, Fig. S3C and D, upper panels). However, *Acanthamoeba* cells infected with 60-min UV-inactivated *Marseillevirus* particles exhibited partial retraction of acanthopodia, with some changes in cell morphology even though no VFs formed (Fig. 4C). Moreover, cells infected with 30-min UV-inactivated *Marseillevirus* particles showed CPE, but no VF formation (Fig. S3A to C, lower panels). Collectively, these data indicated that rounding of cells in *Mimivirus* infection requires VF formation and viral protein synthesis, whereas internalization of virus particles is sufficient to induce CPE in *Marseillevirus* infection.

Quantitative analysis further revealed that compared to that in uninfected cells, at 8 hpi, 12-min UV-irradiated *Mimivirus* showed about 8% and 18% reductions in cell size and acanthopodia number (Fig. 4D and E); while 60-min UV-irradiated *Marseillevirus*-infected cells showed about 28% and 30% reductions in cell size and acanthopodia number (Fig. 4F to G). In addition, 30-min UV-irradiated *Marseillevirus*-infected cells showed about 45% and 28% reductions in cell size and acanthopodia number (Fig. 4F to G). This further indicates that UV treatment of *Marseillevirus* for 30 and 60 min abolished VF formation upon infection, but after 60 min of UV treatment, *Marseillevirus* still remained partially active but could not form VF.

**FIG 5:**
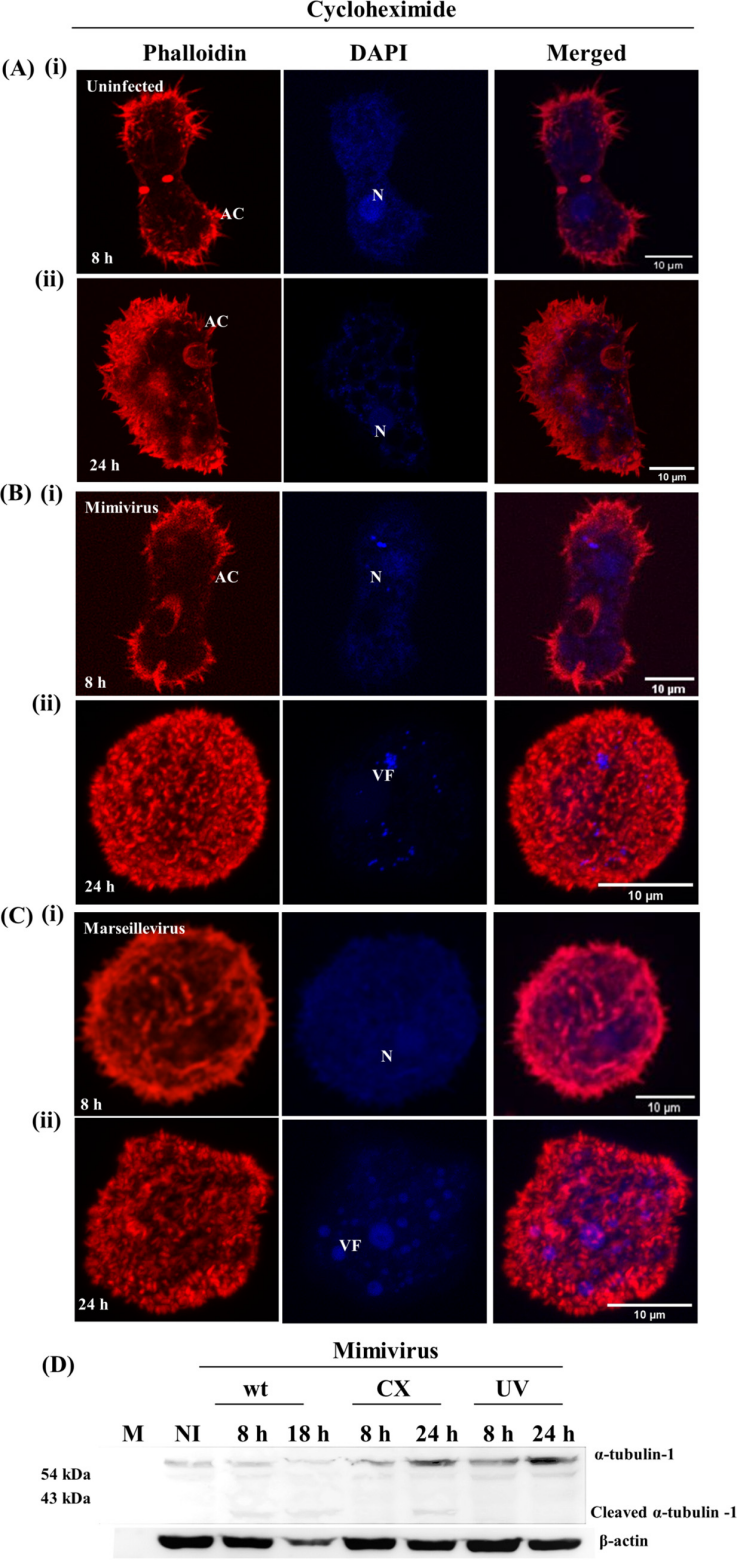
Virus protein synthesis is not essential for *Marseillevirus*-induced CPE. Cells were fixed and then stained with Alexa Fluor 555-labeled phalloidin (red), counterstained with DAPI (blue), and analyzed using confocal microscopy at 8 (i) and 24 hpi (ii). (A) Cycloheximide-treated uninfected A. castellanii cells, (B) Cycloheximide-treated *Mimivirus*-infected A. castellanii cells, (C) Cycloheximide-treated *Marseillevirus*-infected A. castellanii cells. VF, viral factory; N, nucleus; AC, acanthopodia. Scale bar = 10 μm. (D) Western blot analysis of cell lysate collected from *Mimivirus*-infected cells under indicated treatment conditions with anti-tubulin antibody. Actin was used as a loading control.

To determine whether the viral proteins produced in the VF are essential for CPE, A. castellanii cells were treated with cycloheximide, a well-known protein synthesis inhibitor, 15 min prior to infection with *Mimivirus* and *Marseillevirus* particles. Cycloheximide concentration was optimized for A. castellanii cells. We found that a 30 μM concentration of cycloheximide was not toxic and did not affect the growth of AC cells, and hence we used this concentration for all experiments (Fig. 5A, panels i and ii). Infection of cycloheximide-treated AC cells with *Mimivirus* did not produce any CPE until 8 hpi (Fig. 5B, panel i). By 24 hpi, cells exhibited rounding, suggesting that the *Mimivirus* infection cycle was delayed (Fig. 5B, panel ii). In contrast, no such time delay in CPE was observed when cycloheximide-treated cells were infected with *Marseillevirus*, although VF formation and cell lysis were delayed to 24 h (Fig. 5C, panels i and ii). A further delay in infection in cycloheximide-treated *Mimivirus*-infected cells was confirmed by Western blot analysis. Lysate collected from cycloheximide-treated *Mimivirus*-infected cells at 24 h showed the presence of cleaved α-tubulin comparable to lysate from *Mimivirus*-infected cells at 8 h. In contrast, a cleaved tubulin band did not appear even after 24 h in UV-inactivated *Mimivirus*-infected cells (Fig. 5D). These results suggest that in the case of *Marseillevirus*, the prepackaged proteins are sufficient to induce CPE during infection, while *Mimivirus* may require the synthesis of early/late proteins to establish a productive infection.

### *Mimivirus* early/late proteins mediate α-tubulin cleavage and depolymerization of actin late in infection.

**FIG 6:**
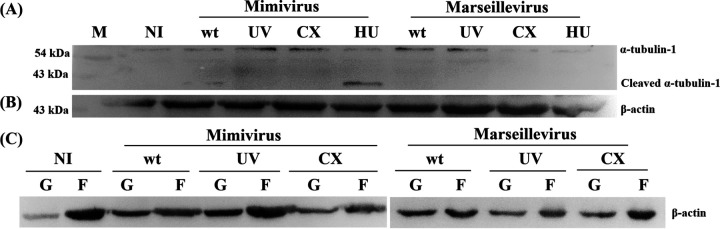
Expression of early/late viral proteins is essential for actin depolymerization and tubulin cleavage during *Mimivirus* infection. Uninfected (NI), *Mimivirus*-infected, and *Marseillevirus*-infected cells under indicated treatment conditions were collected at 8 hpi. The amount of tubulin (A) or actin (B) and changes in actin dynamics (C) in cell lysates were determined using Western blot analysis with anti-actin and anti-tubulin antibodies. Three independent experiments were performed.

Because depolymerization of actin and cleavage of tubulin appear to be the major factors in bringing on CPE, we tested for tubulin cleavage and actin depolymerization after 8 hpi in cycloheximide-treated AC cells and AC cells infected with UV-treated *Mimivirus* and *Marseillevirus* particles. Tubulin cleavage was not observed when cycloheximide-treated AC were used as host cells for *Mimivirus* infection. Similarly, UV-treated *Mimivirus*-infected cells failed to show the presence of cleaved tubulin (Fig. 6A). While total actin levels remained the same in all cases (Fig. 6B), under the conditions where no CPE was observed (UV-treated virus particles), actin depolymerization (as determined by the ratio of G-actin to F-actin) was not observed. In contrast, UV-inactivated *Marseillevirus*- and wild-type *Marseillevirus*-infected cells exhibited comparable levels of F-actin and G-actin (Fig. 6C). This further confirms that synthesis of *Mimivirus* early/late proteins is essential for tubulin cleavage and actin depolymerization to induce CPE.

We also attempted to investigate the effect of actin stabilization on CPE by using the drug jasplakinolide. We found that at concentrations of up to 2 μM, there was no change in virus-induced CPE compared to that in the control (Fig. S4C), while slightly higher concentrations of the drug (4 μM) were found to be toxic in uninfected *Acanthamoeba*.

## DISCUSSION

This study was designed to gain insights into early host modification during the infection of Acanthamoeba castellanii cells by *Mimivirus* and *Marseillevirus*. Cytoskeleton, which governs cellular processes such as cell shape, phagocytosis, cell movement, and organelle distribution, is a common target exploited by many pathogens, including *Mimivirus* and *Marseillevirus*, to facilitate their entry and establish infection (49–51). Previous studies have shown that the replicative cycles of *Marseillevirus* and *Mimivirus* take about 5 and 8 h, respectively. The release of new progenies of *Marseillevirus* begins at 24 h either as single particles or as vesicles enclosing several particles through lysis of the infected host cell, while progeny *Mimivirus* particles are released only as individual particles at about 12 hpi. *Mimivirus*-infected rounded cells showed retracted acanthopodia and fragmented microtubules (12, 41). Consistent with this observation, our live-cell imaging showed significant changes in cell morphology and cytoskeletal rearrangement between 4 and 6 hpi during *Mimivirus* infection. By 8 hpi, when the late viral proteins are predominantly synthesized, *Mimivirus*-infected cells not only acquired a completely rounded morphology but also showed the characteristic tubulin cleavage (Fig. 1 and 2).

In contrast, most of the *Marseillevirus*-infected cells acquired a partially rounded morphology relatively early during the infection (~4 hpi, Fig. 1 and 3). *Marseillevirus*-infected cells also exhibited partial retraction of acanthopodia and rearrangement of microtubules earlier than *Mimivirus*-infected cells. The characteristic cleavage of tubulin during the later stages of *Mimivirus* infection is consistent with the appearance of the fragmented microtubule structure after 6 hpi, while the *Marseillevirus*-infected cells only showed a decrease/rearrangement in the microtubule loops but showed neither fragmentation nor cleavage of tubulin at any stage of infection (Fig. 1 and 2, Fig. S2). The retraction of acanthopodia (due to depolymerization of actin) was commonly observed in both *Mimivirus*- and *Marseillevirus*-infected cells (Fig. 1 and 2, Fig. S2).

Interestingly, during the infection of *Acanthamoeba* cells with UV-treated *Mimivirus* and the treatment of *Mimivirus*-infected *Acanthamoeba* cells with cycloheximide, CPE, including actin depolymerization, tubulin cleavage, and fragmented microtubules, were not detected (Fig. 4 to 6). This clearly suggests that the synthesis of viral transcripts and proteins is essential for bringing about CPE. Omics studies have suggested that both *Mimivirus* and *Marseillevirus* particles package several proteins and a few translation-ready transcripts inside the capsid (14, 52). We attempted to distinguish between the roles of *Mimivirus* early and late proteins by treating infected cells with hydroxyurea, an inhibitor of viral DNA replication (1). Because hydroxyurea was found not to inhibit both *Mimivirus* and *Marseillevirus* DNA replication even at very high concentrations (Fig. S4A), we could not ascertain the roles of early and late Mimiviral proteins in actin depolymerization and tubulin cleavage. Nevertheless, induction of CPE only after 6 h of infection strongly suggests that *Mimivirus*-induced CPE is most likely a post-replicative event brought about by the newly synthesized proteins and not by the packaged proteins or transcripts.

Surprisingly, even in the absence of viral factory formation upon DNA damage due to 30-min UV treatment (Fig. S3A to C), *Marseillevirus* particles could still induce CPE, including the changes in cell morphology and depolymerization of F-actin (Fig. 4C, 5C, and 6C). Because UV exposure blocked both replication and transcription, as also seen in the case of the VV (53), it is likely that proteins which are packaged in the capsid are responsible for inducing CPE in *Marseillevirus* infection. As in the case of *Marseillevirus*, the vaccinia virus-induced CPE is a pre-replicative event, but in VV-infected cells, the CPE is mediated by early viral proteins (1, 53). While the changes in actin filament arrangement in response to external stimuli results in rapid changes to cell morphology, microtubule arrangement largely appears to be a function of the morphological state of amoeba (54–56). Hence, we speculate that *Mimivirus* and *Marseillevirus* proteins most likely target the host actin filaments rather than microtubules to induce CPE. For instance, during *Legionella* infection, several unique pathogen-encoded genes are involved in cytoskeletal rearrangement; for example, LegG1 promotes microtubule stabilization via activating Ran GTP, but LegK2, a protein kinase, inhibits actin polymerization on the *Legionella*-containing vacuole (27, 57, 58).

While actin depolymerization has been widely reported with many intracellular pathogens, such as *Yersinia*, Salmonella, and herpes simplex virus (59–64), to the best of our knowledge, intracellular pathogen-mediated cleavage of tubulin has not been reported so far. *Legionella*, an intracellular bacterial pathogen that infects protozoan and human hosts, has been reported to cause similar rounding of amoebal cells during infection, and some of the components responsible for bringing about CPE have been identified and characterized (25–28, 49, 65). A recent study reported the *in vitro* cleavage of actin by a Zn metalloprotease encoded by *Legionella*. However, *in vivo* cleavage of actin during *Legionella* infection of *Acanthamoeba* was not observed (28). Considering the role of the Zn metalloprotease of L. pneumophila in the *in vitro* cleavage of actin, we hypothesized that a similar protein could be involved in tubulin cleavage during *Mimivirus* infection. The *Mimivirus*-encoded Zn metalloprotease gp560 (product of gene R519) belongs to the M13 family of Zn metalloprotease containing all the conserved motifs (Fig. S5A to B). We found that while the proteolytic activity of the recombinant gp560 protein was evident on the gelatin substrate, the purified protein failed to depolymerize actin and cleave tubulin *in vitro* under all conditions tested (Fig. S5).

Although there are no reports on tubulin cleavage during infection, caspase-mediated tubulin and actin cleavage for axonal degeneration have been reported. In most of these cases, tubulin cleavage has been reported to be mediated by caspase 6 (cysteine-aspartic protease), granzyme M, and granzyme B (serine proteases) at the L269 or D438 residue. Additionally, at least one other cleavage site is also present C-terminal to L269. Protease-induced concomitant morphological changes due to cytoskeleton degradation are known to be associated with the rupture of host cell membrane/cell death (47, 66–69).

Our live-cell imaging showed that while *Mimivirus*-infected cells release new progenies instantly by bursting host cells, between 12 and 24 hpi, *Marseillevirus*-infected cells released viruses mostly as aggregates, as shown previously using fixed cells (12–14), but no rapid bursting of the infected cell as seen in the case of *Mimivirus* (compare Movies S3 and S4 to S5) was observed. Similar to *Mimivirus* release, Chlamydia release has also been reported by two mutually exclusive pathways, spontaneous release by rupturing of the plasma membrane and slow extrusion. It was further reported that the lysis of the plasma membrane is not due to mechanical stress exerted on the cell, but instead is an ordered sequence of membrane permeabilization which is completely impaired by the inhibition of intracellular calcium signaling (69). Additionally, Chlamydia induces caspase-independent apoptosis due to the overexpression of BAX at the end of infection cycle to mediate release of Chlamydia. However, Chlamydia-infected BAX-deficient cells die *in vitro* through necrosis instead of apoptosis (70–72). Although apoptosis in *Acanthamoeba* has also been reported, unlike in mammalian cell infection, *Legionella* could not induce apoptosis in *Acanthamoeba* (73–76). Nevertheless, late during infection, *Legionella* induces necrotic cell death in *A. polyphaga* for the egress of bacteria upon termination of bacterial proliferation (77–79). The marine sponge *Aaptos aaptos* can induce both apoptotic and necrotic cell death in *Acanthamoeba* (80). Disassembly of the actin cytoskeleton is considered one of the events during the apoptosis-like process in *Acanthamoeba* (81). Apoptosis-like cell death was also seen at the end of the Salmonella infection cycle in *A. rhysodes*, probably to mediate the release of Salmonella (82). On the other hand, increased internal pressure due to the accumulation of progeny virus particles is sufficient for the rapid bursting of cells as generally seen in lytic bacteriophages (83). Taking our findings and literature together, we hypothesize that tubulin cleavage along with actin depolymerization might provide a signal to host *Acanthamoeba* either for apoptotic or necrotic cell death for the release of progenies through the bursting of the cell. It is also possible the mechanical instability caused by cytoskeletal modifications and accumulation of progeny viruses could cause rapid bursting of cells in *Mimivirus*-infected AC cells.

Finally, we found that the *Mimivirus*-coded Zn metalloprotease is unable to cleave the commercially available porcine brain tubulin *in vitro*. Porcine brain tubulin shares about 70% sequence identity with tubulin from A. castellanii. Hence, we cannot rule out the possibility of the role of *Mimivirus*-encoded serine/cysteine proteases in host tubulin cleavage. The *Mimivirus* genome encodes several proteases, including Zn metalloprotease, serine, and cysteine proteases (gp274, gp563, gp512, gp384, gp560, and gp255). Further systematic studies are needed to delineate the roles of *Mimivirus* and *Marseillevirus* encoded factors in the depolymerization of actin.

Recently, it was reported that some members of the viral order *Imitevirales*, such as *Yasminevirus* of the *Klosneuviridae* subfamily of the phylum *Nucleocytovircota* (the NCLDVs) code for an actin-like protein called viractin (84). Although the role of viractin in the viral life cycle and/or hijacking of the host is not clear, the presence of viractin mRNA in the transcriptomes of plankton (from the same sample) suggested that the viractin gene is expressed. Extensive phylogenetic analysis has further suggested that the viractin was recruited, early probably from a proto-eukaryotic host, suggesting a coevolution of the Last Eukaryotic Common Ancestor (LECA) and its viruses that could have shaped the modern day cytoskeleton. Functional and structural characterization of viractin would help to establish its role in the viral life cycle.

**FIG 7:**
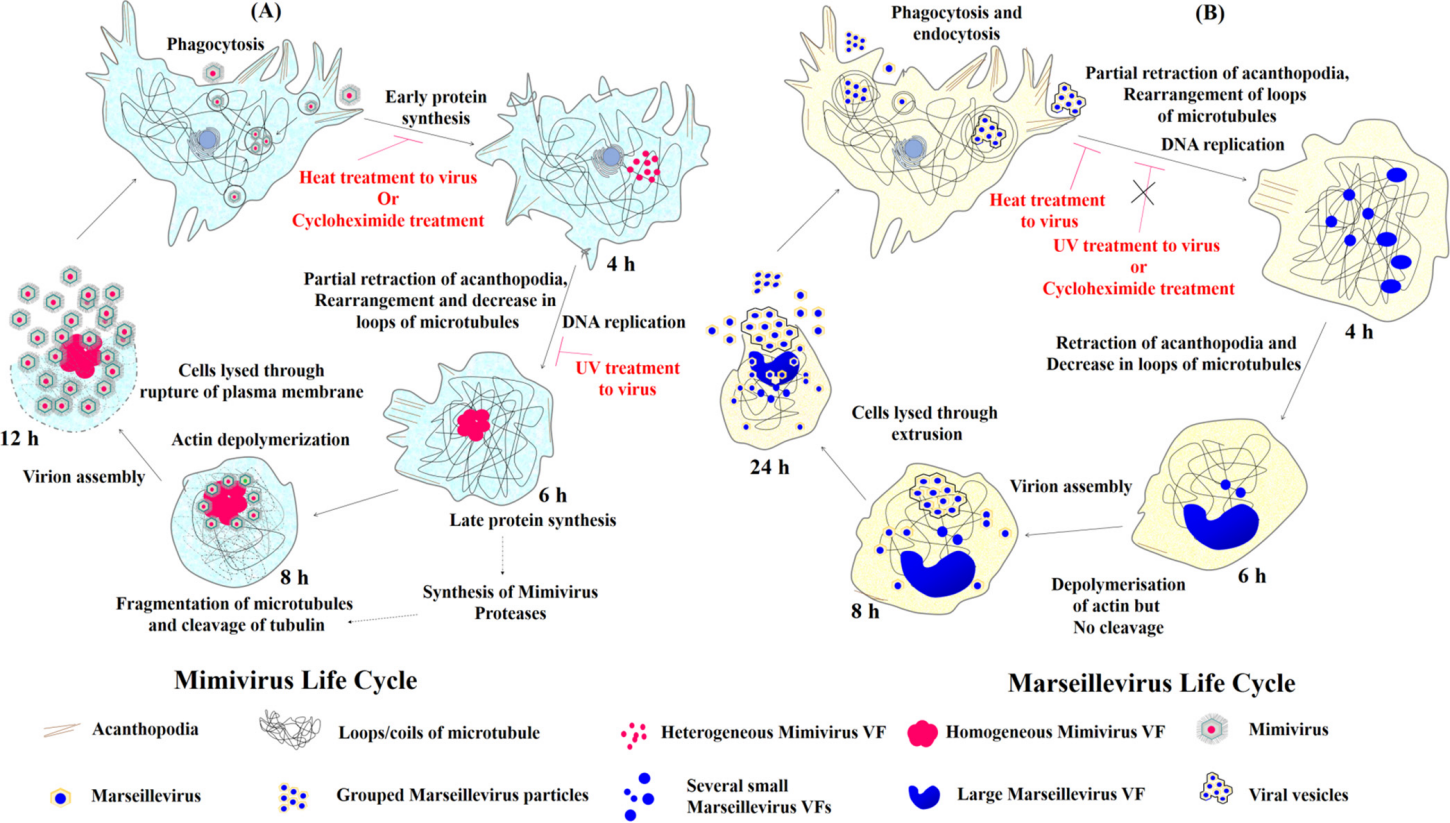
Overview of the *Mimivirus* and *Marseillevirus* life cycle and sequence of motile events described in this study.

Based on the findings from this study as well as what was already known, we have proposed an updated model for the replication cycles of *Mimivirus* and *Marseillevirus* (Fig. 7). *Mimivirus* enters through phagocytosis, while *Marseillevirus* enters via phagocytosis and endocytosis. During *Mimivirus* infection, no significant change in cell morphology and the arrangement of acanthopodia and microtubules were observed until about 4 hpi, and several small heterogeneous VFs were visible between 4 and 6 hpi. While the smaller VFs merged to form a single mature VF between 6 and 8 hpi in most infected cells, the presence of two mature, productive VFs was also seen consistently in some infected cells. After about 6 hpi, the synthesis of late proteins begins with a concomitant decrease in actin filaments and fragmentation of microtubule, presumably as consequences of actin depolymerization and tubulin cleavage, respectively. By 8 hpi, the presence of fully assembled progeny viral particles can be seen around the VF (Fig. 7A).

In contrast, *Marseillevirus*-infected cells exhibited CPE relatively early and exhibited several small mature VFs. Between 6 and 8 hpi, the presence of progeny viral particles was observed, but tubulin cleavage/microtubule fragmentation did not occur. Both *Mimivirus*- and *Marseillevirus*-infected cells remain rounded until cell lysis. UV- or heat-inactivated *Mimivirus*-, heat-killed *Marseillevirus*-, and cycloheximide-treated *Mimivirus*-infected cells, but not UV-inactivated *Marseillevirus*- or cycloheximide-treated *Marseillevirus*-infected cells, were able to thwart virus-induced CPE (Fig. 7A and B).

In summary, the characteristic cleavage of α-tubulin during *Mimivirus* infection appears to be novel and has not been seen during any other intracellular pathogen infection. The *in vivo* role of *Mimivirus*-encoded proteases in tubulin cleavage, as well as whether tubulin cleavage and actin depolymerization are associated with apoptotic or necrotic cell death during *Mimivirus* infection, need to be further investigated.

## MATERIALS AND METHODS

### Culture of *Acanthamoeba* and virus infection.

A. castellanii was cultured in T-25 flasks in 4 mL of PYG medium axenic conditions until it reached 80% confluence and was then infected with *Mimivirus* (MOI = 10) or *Marseillevirus* (MOI = 10) (38).

### Inactivation of virus particles.

For UV treatment, suspensions of *Mimivirus* and *Marseillevirus* (MOI = 100) were incubated for 12 and 60 min, respectively, at a 30-cm distance from the laminar UV light (254 nm). For heat treatment, suspensions of *Mimivirus* and *Marseillevirus* were incubated at 70°C for 15 min. *Mimivirus* and *Marseillevirus* were labeled with SYBR Green I (Sigma-Aldrich, cat no. S7563) by a modification of the method used for staining *E coli* (85). Briefly, virus lysate was centrifuged at 8,000 × *g* for 30 min to pellet the virus particles. The pellet was washed with phosphate-buffered saline (PBS) and centrifuged again. The obtained pellet was incubated in SYBR Green I (Sigma-Aldrich, 10,000× diluted), washed, and stored in PBS at −20°C.

### Immunofluorescence microscopy.

*Mimivirus*- and *Marseillevirus*-infected *Acanthamoeba* cells were grown on coverslips, fixed with a combination of paraformaldehyde and glutaraldehyde, washed, permeabilized, and treated with Alexa Fluor-labeled 555 labeled phalloidin (Invitrogen, cat no. A34055), rat α-tubulin monoclonal antibody MAb (yeast; Bio-Rad, cat no. MCA78G), and incubated with Alexa Fluor 488-conjugated goat anti-rat polyclonal IgG antibody (Jackson, cat no. 112-545-167). Cells were counterstained with DAPI (4′,6-diamidino-2-phenylindole) and then mounted on glass slides.

### Live cell imaging.

For continuous monitoring of the initial steps of virus infection, the motility of *Acanthamoeba* cells was reduced using 1% low melting agarose. Briefly, *Acanthamoeba* cells were infected with SYBR Green I-stained virus and then cultured with 1% low melting agarose containing PYG medium. To monitor the lysis of virus-infected cells and examine the morphology of cells infected with UV- and heat-inactivated virus particles, cells were cultured in PYG medium without low-melting agarose. The differential interference contrast (DIC) images were captured using a Carl Zeiss LSM 780 microscope (Zeiss, Germany). Videos of *Acanthamoeba* cell lysis during infection were recorded using a combination of the ‘tile scanning’ and ‘time series’ options available in the Zeiss microscopy software (ZEN black edition). The tiling and stitching options were used to generate high-resolution images covering a larger area of multiple continuous fields. The videos were processed with Zeiss microscopy software (ZEN blue edition, available online). For this, a ‘region of interest’ was drawn in the video using the ‘ROI’ tool under ‘Graphics’ to create a subset of the selected video, which helps display the lysis of a single cell in an image (or video). ImageJ was used to generate the movies, and the videos were further converted into images using ZEN blue software.

### Quantification of cell size, acanthopodia number, and microtubule dynamics.

To determine the approximate sizes, the area occupied by different cells on the cover slip was drawn manually using the freehand selection tool available in Zen blue software, as described previously (1). The border of each cell was defined by phalloidin staining. Polymerized tubulin in infected cells was quantified using the filament sensor software designed for the estimation of actin stress fibers (86). The filopodia number in each cell was quantified using the FiloQuant plugin in ImageJ Java software as described previously (87). Briefly, processed images were imported into ImageJ and to the filament sensor. To determine the standard settings to detect the number of acanthopodia both within and at the periphery of each cell, each cell was outlined by hand to select the region of interest (ROI) and then different steps were performed as suggested by the manual (87). Depending on the threshold of each cell, these settings were used with some further modifications for the analysis of acanthopodia in infected and uninfected cells at different times of infection. For polymerized tubulin quantification, each cell was outlined by hand to extract a single cell image. Contrast was adjusted for each image before quantification and each step was followed as mentioned by the author (86). Polymerized tubulin for each cell was determined by calculating the integrated pixel count of regions of interest.

### Treatment of *Acanthamoeba* cells with cycloheximide.

Cycloheximide (Sigma-Aldrich, cat no. C7698) was maintained as a stock solution of 0.4 M and stored at 4°C. Trophozoites were pretreated with cycloheximide at a 30-μM final concentration for 15 min, infected with *Mimivirus* and *Marseillevirus* for 8 and 24 hpi, and subsequently used for confocal imaging. The drug does not cause cell death and delays amoebal division by about 24 h.

### Western blotting.

*Mimivirus*- and *Marseillevirus*-infected *Acanthamoeba* cells (MOI = 10) were washed in PBS and then resuspended in microtubule stabilization buffer (0.1 M PIPES [pH 6.9], 2 mM EGTA, 2 mM ATP, 5 mM MgCl_2_, 10% glycerol, 0.5% Triton X-100, and protease inhibitor cocktail). For differential extraction of actin monomers and polymers from cells, a protocol was followed as described previously for mammalian cells with slight modifications (57, 88). Briefly, cells were lysed, an equal volume of lysed cells was collected, and the remaining part was centrifuged at high speed (35,000 × *g* for 1.5 h) to separate the F-actin in the pellet fraction and the G-actin in the supernatant fraction. The pellet containing F-actin was resuspended in 8 M urea. The protein concentration was determined, and identical concentrations of protein were loaded in each well and then separated by SDS-PAGE gel. The membrane was blotted with an anti-actin (Invitrogen, cat no. ACTN05, C4, MA5-11869) and anti-tubulin (yeast, Bio-Rad, cat no. MCA78G) antibodies and with secondary antibody conjugated to horseradish peroxidase. Blot images were captured using the ChemiDoc gel imaging system (UVitech, USA). ImageJ was used to measure the intensity of bands corresponding to actin and tubulin. The ratio of F- to G-actin revealed the amount of actin depolymerization during *Mimivirus* and *Marseillevirus* infection.

### Statistical analysis.

Statistical analysis data were represented as means ± SD (Fig. 1, 2, and 4) derived from three independent experiments. Comparisons between two groups were analyzed using a two-way analysis of variance (ANOVA) (Fig. 2). One-way ANOVA was used for comparisons within a group (Fig. 1 and 4), with *P* < 0.05 were considered statistically significant. Graphs were constructed using GraphPad Prism version 6.

### Data availability.

All data from the study have been submitted.
