# Supplementary material for: Amoebal Tubulin Cleavage Late during Infection Is a Characteristic Feature of Mimivirus but Not of Marseillevirus
Source: Microbiol Spectr. 2022 Dec 1;10(6):e02753-22. doi: 10.1128/spectrum.02753-22 (PMC9769910; doi:10.1128/spectrum.02753-22)
Supplement: Supplemental file 1 — Supplemental material. Download spectrum.02753-22-s0001.pdf, PDF file, 1.0 MB [file spectrum.02753-22-s0001.pdf]

Amoebal Tubulin Cleavage Late during Infection is a Characteristic Feature of Mimivirus but not of Marseillevirus

Nisha Goyal, Amlan Barai, Shamik Sen and Kiran Kondabagil*

Supplemental data

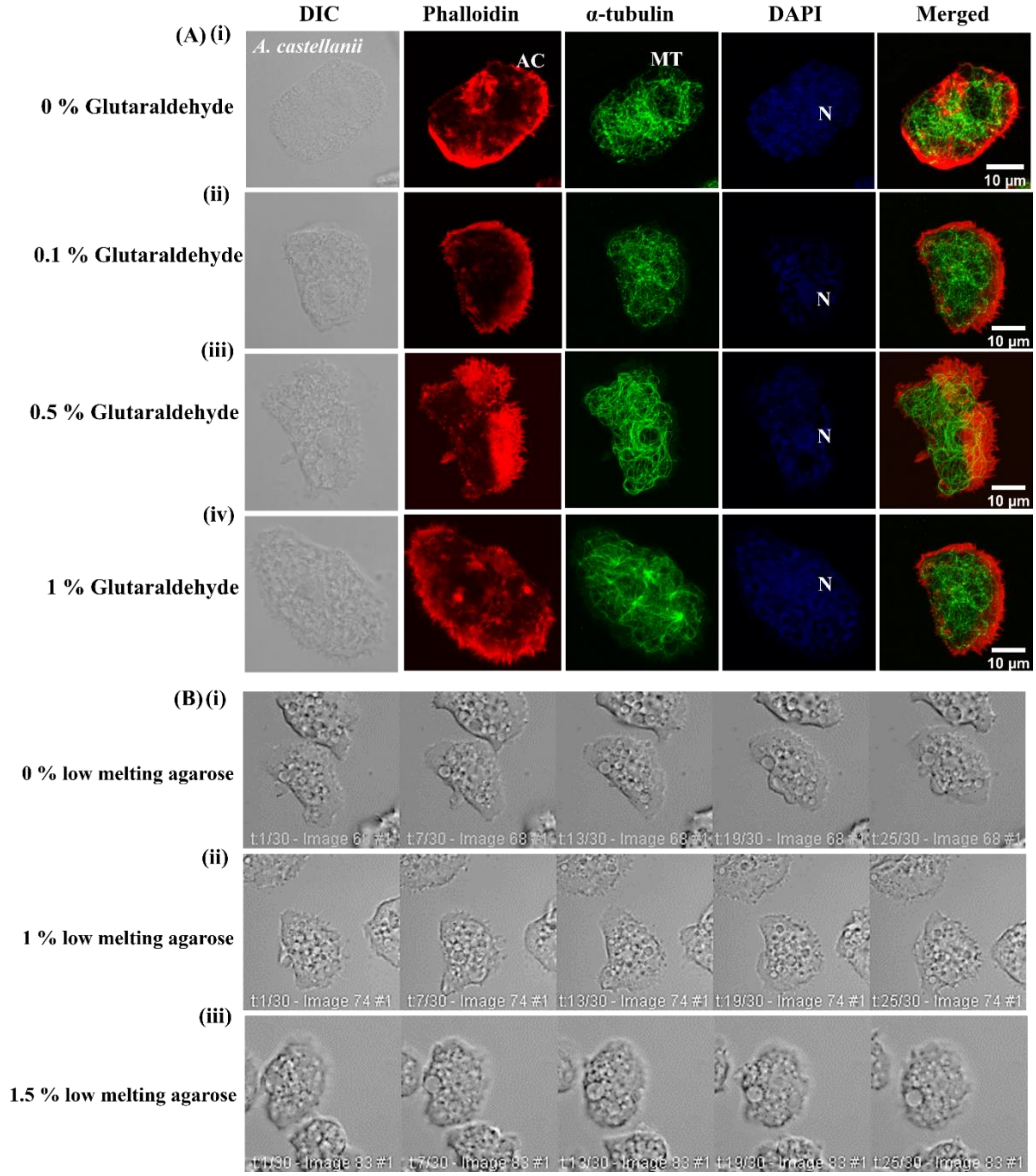

FIG S1. Optimization of protocol for confocal and time-lapse microscopy of *A. castellanii*. (A) *A. castellanii* was cultured on the cover slips, fixed with paraformaldehyde in combination with different concentrations of glutaraldehyde. *Acanthamoeba* cells were fixed (i) with 4 %

paraformaldehyde alone or (ii) with 3.9 % paraformaldehyde and 0.1 % glutaraldehyde. The morphology of cells as well as of cytoskeletal network and nucleus was not preserved. *Acanthamoeba* cells were fixed (iii) with 3.5 % paraformaldehyde and 0.5 % glutaraldehyde or (iv) with 3 % paraformaldehyde and 1 % glutaraldehyde. The loops of microtubules as well as acanthopodia at cell surface were clearly visible. N, nucleus. AC, acanthopodia. MT, microtubules. Scale bar, 10 µm. (B) To monitor the changes in cell morphology early during infection, a protocol was optimized for the time-series experiments. *Acanthamoeba* cells were cultured in PYG media alone (i), containing 1 % low melting agarose (ii), and 1.5 % low melting agarose (iii). The motility of *Acanthamoeba* was reduced in the presence of 1 % low melting agarose thus used for monitoring change in the cell morphology early in infection. *Acanthamoeba* cells were observed using confocal microscopy for 1.5 min at 3 sec intervals for monitoring the motility.

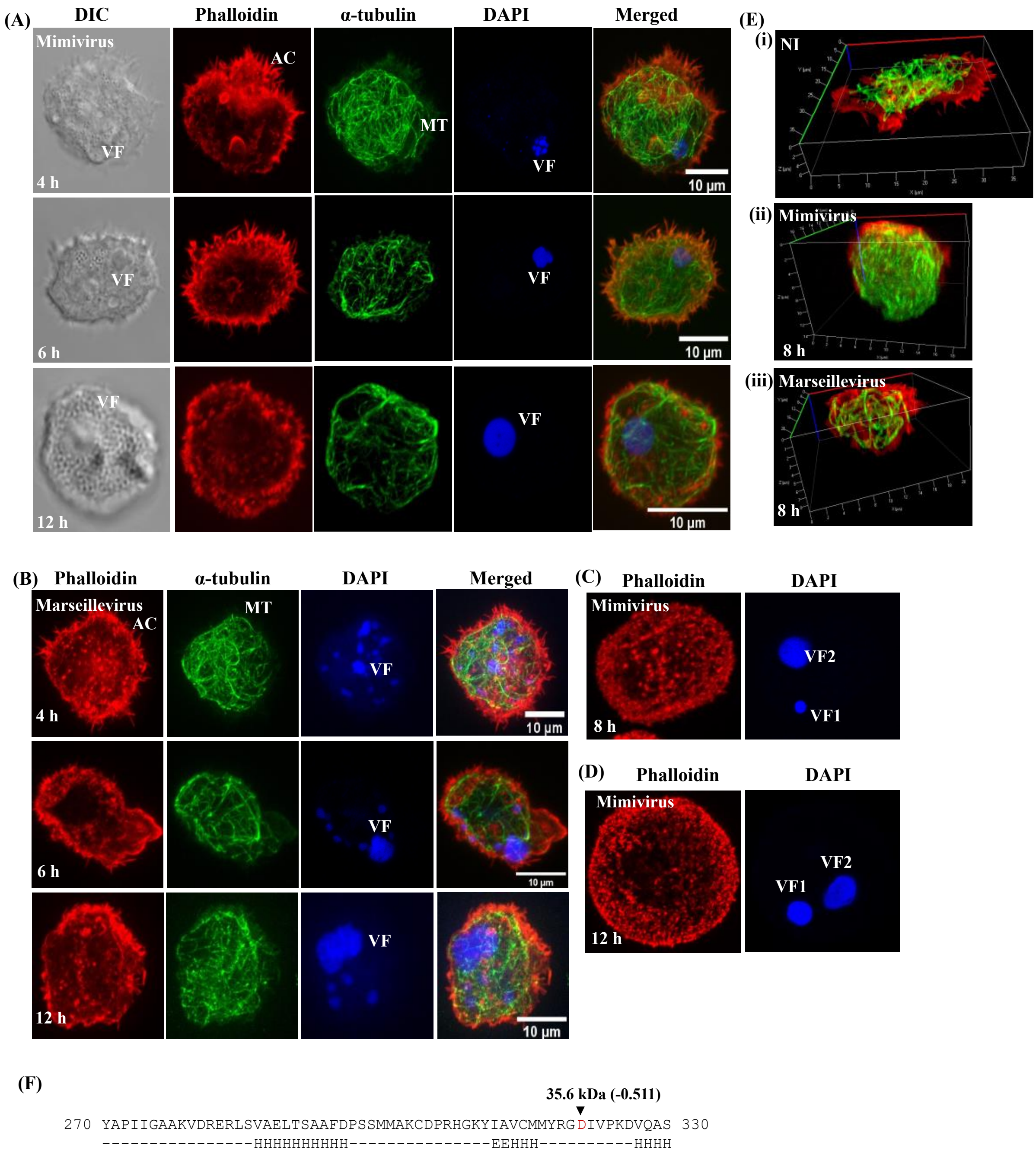

S2 Figure. Host cytoskeletal modifications during Mimivirus and Marseillevirus infection. Mimivirus and Marseillevirus infected *Acanthamoeba* cells were stained with Alexa Fluor 555 labelled phalloidin (red), *Yeast* α-tubulin antibody (Green) and counterstained with DAPI (blue) at 4 h, 6 h, 8 h and 12 h pi. (A) At 4 h pi, Mimivirus infected *Acanthamoeba* cells exhibited several small VFs at a distinct location, while (B) Marseillevirus infected cells showed several small VFs throughout in the cytoplasm. Interestingly, individual Marseilleviral factories further did not fuse with each other even till 12 h and thus, appeared as large and diffusive VF during Marseillevirus infection. (C, D) Two VFs were formed in small fraction of Mimivirus infected cells only at 8 h and 12 h pi. One factory was comparatively bigger than the other one. Both of them did not fuse with each other even late during infection (about 12 h). (E) 3-D images showing cell area as well as cell height in uninfected (i), Mimivirus infected (ii) and Marseillevirus

infected cells (iii) at 8 hpi. VF, homogeneous viral factory. VF1, small viral factory. VF2, large viral factory. N, nucleus AC, acanthopodia. MT, microtubules. Scale bar, 10 µm. (F) Predicted cleavage site on the α-tubulin-1 of *Acanthamoeba castellanii*. Hydrophobicity of Aspartate residue measured using Expasy tool was shown in bracket. The molecular mass of the α-tubulin-1 fragment after the cleavage at the predicted D^{321} site is about 36 kDa.

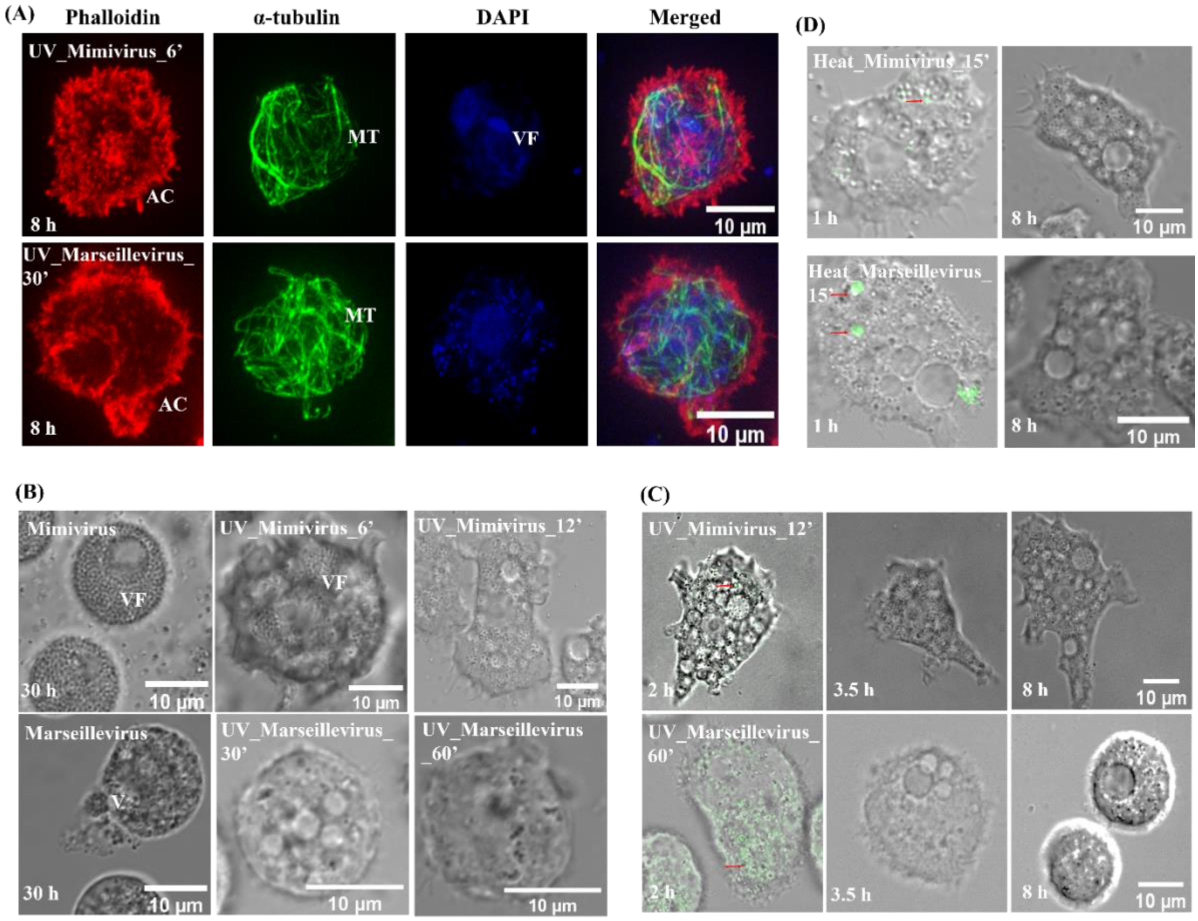

FIG S3 Confocal and time lapse microscopy confirms inactivation of viral particles with UV and heat treatment. AC cells infected with UV irradiated Mimivirus or Marseillevirus were observed using confocal microscopy at 8 h (A), and 30 hpi (B). AC cells infected with 6 min UV irradiated Mimivirus (upper panels of A) showed smaller sized VFs at 8 hpi. Lysis was delayed in cells infected with 6 min UV irradiated Mimivirus (upper panels of B) but did not occur in cells infected with 12 min UV irradiated Mimivirus as well as in 30 min and 60 min UV irradiated Marseillevirus infected cells. (C) Time lapse microscopy of AC cells infected with SYBR Green I stained UV inactivated Mimivirus or Marseillevirus. Infected cells were observed from 2-8 hpi. (D) AC Cells infected with SYBR Green I stained heat-killed Mimivirus or Marseillevirus at 8 hpi. SYBR Green I stained UV inactivated or heat-killed Mimivirus and Marseillevirus has the ability to infect the host cells (red arrows). VF, viral factory; MT, microtubules; V, new progenies; AC, acanthopodia. (Scale bar, 10 μm).

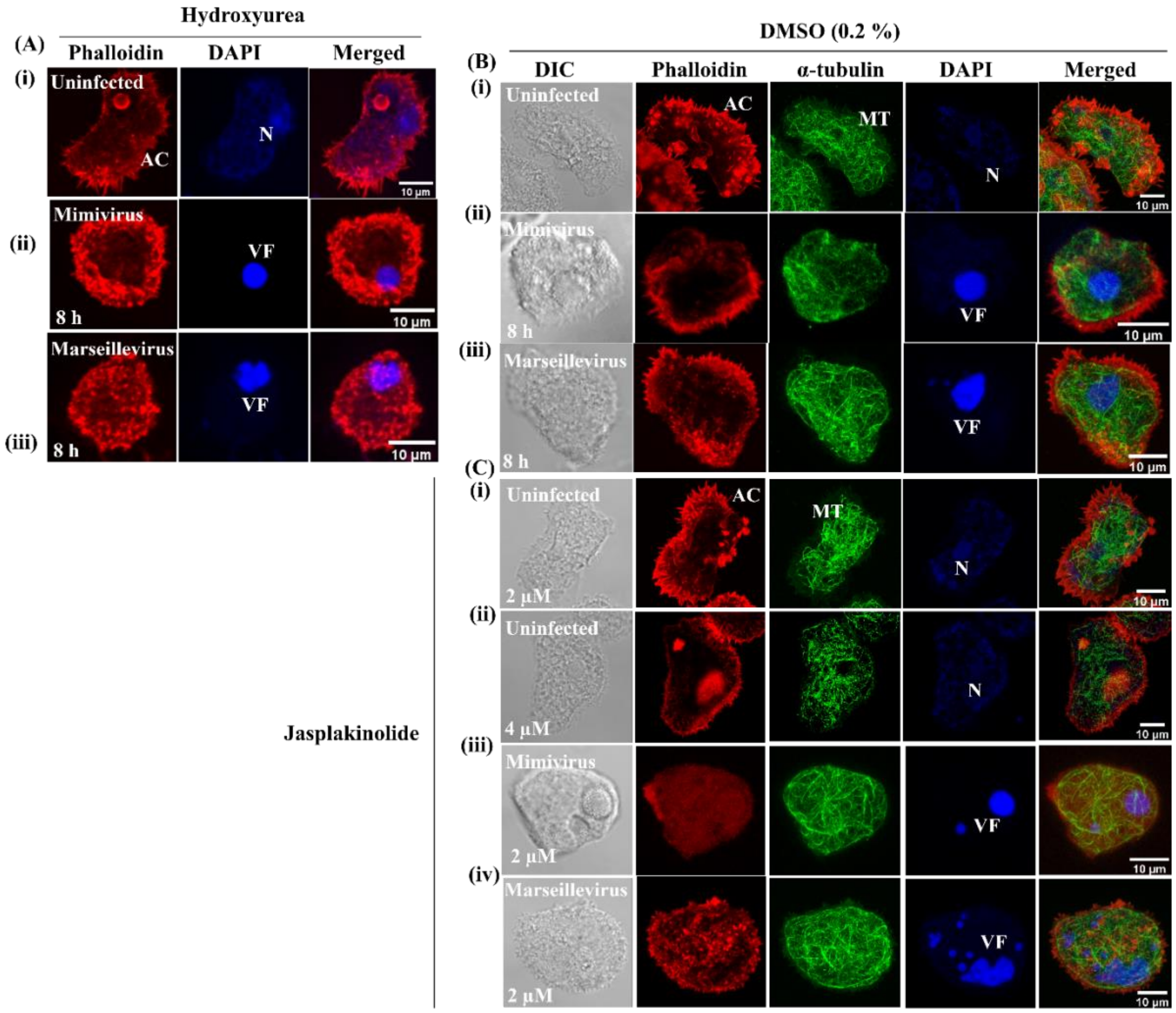

FIG S4 Effect of drug hydroxyurea/jasplakinolide treatment during Mimivirus and Marseillevirus infection. (A) Stock concentration of hyroxyurea drug (1.3 M) was prepared freshly in sterile water. Trophozoites were pre-treated with hydroxyurea at 2 mM final concentration for 15 min and then infected with Mimivirus or Marseillevirus at an MOI of 10. Subsequently, imaging was done using confocal microscopy at 8 hpi. (i) The drug did not show toxic effect on uninfected *Acanthamoeba*. (ii, iii) Hydroxyurea-treatment did not affect Mimivirus and Marseillevirus infection of AC cells. (B, C) To assess whether change in cell morphology during infection is essential for VF formation, virion assembly or cell lysis; *Acanthamoeba* trophozoites were treated with jasplakinolide (1 mM stock solution, sigma, 420127), 20 min after Mimivirus or Marseillevirus infection and observed under confocal microscope at 8 hpi. (B) As a vector control, uninfected (i), Mimivirus infected (ii) and Marseillevirus infected (iii) AC cells were treated with 0.2 % DMSO. (C) To optimize the concentration of JAS, AC cells were treated with 2 µM (i) and 4 µM (ii). While 4 µM JAS was toxic to AC cells, 2 µM JAS did not affect Mimivirus (iii) and Marseillevirus (iv) infections of AC cells indicating that it is ineffective towards the stabilization of actin filaments of *Acanthamoeba*. VF, viral factory; MT, microtubules; N, nucleus; AC, acanthopodia. (Scale bar, 10 µm).

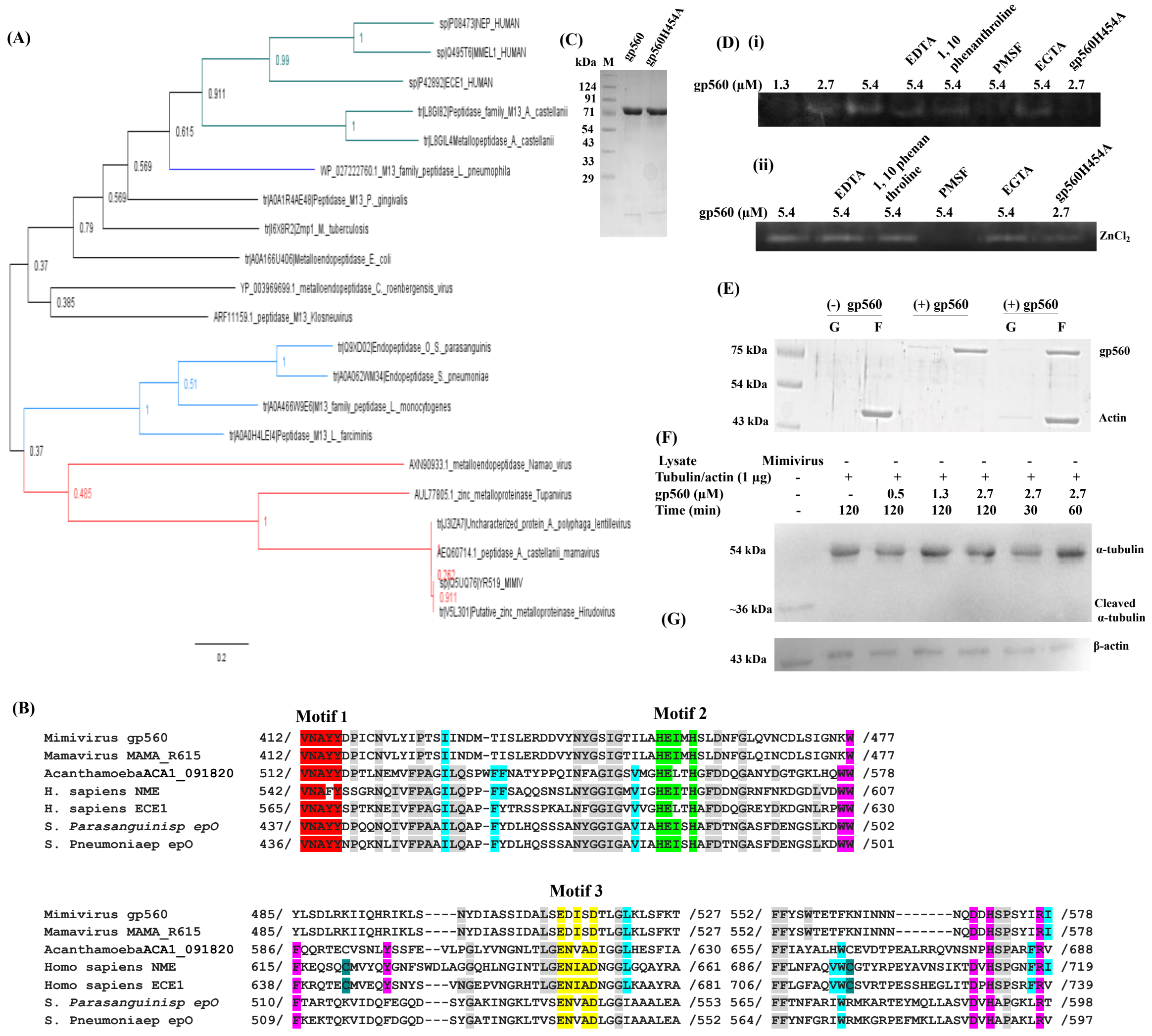

FIG S5. Mimivirus-coded late protein Zn metalloprotease could not induce porcine brain tubulin cleavage. Previously, the role of *Legionella* encoded RavK protein, a Zn metalloproteinase in the cleavage of actin was demonstrated (1). We hypothesized that a similar protease coded by Mimivirus may play a central role in establishing the Mimivirus infection by tubulin cleavage. Search for Zn metalloproteinases containing the signature HExxH motif coded by Mimivirus retrieved two putative Zn metalloproteinases showing 100 % identity with each other suggesting that they are paralogues. Of these, the product of R519 gene (gp560) was found to be a late protein expressed optimally between 6 and 9 hpi and was considered for further analysis.

A BLAST search retrieved a number of homologues of gp560 in pathogenic bacteria, *A. castellanii*, and *Homo sapiens*, as well as in a few members of the Mimiviridae family, but were found to be absent in other NCLDV families. Phylogenetic tree was constructed using Maximum Likelihood method with default settings with 1000 bootstrap replicates in MEGA 6. A phylogenetic reconstruction of the retrieved sequences using Mimiviral gp560 as the seed sequence indicated that putative Zn metalloproteases from some members of the Mimiviridae family forms a cluster with some of the well characterized homologues from bacterial pathogens such as *S. pneumoniae*, and *S. parasanguinis*, although the bootstrap support was low (Fig. S5A). This probably suggests some parallels in the host invasion mechanism between Mimivirus and intracellular bacterial pathogens.

Furthermore, MUSCLE multiple sequence alignment tool was used with default setting to align sequences. A multiple sequence alignment showed that the three signature motifs namely, two Zn binding motifs HExxH (green, motif 2), ExxxD (yellow, motif 3), and the substrate binding motif VNAFY (red, motif 1) as well as other key residues required for the catalytic activity (magenta) and some of the hydrophobic residues associated with S1´ subsite (light blue) that are characteristic of the M13 endopeptidases (2–6) are also conserved in Mimivirus gp560 (Fig. S5B).

To test whether the gp560 protein is involved in the AC cytoskeletal modification, R519 gene was PCR amplified using appropriate primers (Forward 5': GTCCCCCATGGGGATGACATATAGATCATGTATACC-3' and reverse 5'-CGCCCGTCGACATTAATTTTATCTAATATTCG-3'). R519 gene was cloned into the pET28a-His6 vector, transformed into DH5α cells and expressed the corresponding 75-kDa protein with a C-terminal hexa-histidine tag in the E. coli Rosetta (DE3) BL21 competent cells. For this, Rosetta strain carrying pET28a-R519 gene was grown (OD of 0.3-0.4 at 600 nm) in 2 L culture and induced by 0.1 mM IPTG at 25° C for 3 h. The cells were collected, centrifuged at 7000 rpm and the cell pellet was resuspended in 40 ml of lysis buffer (35 mM imidazole, 50 mM Tris HCl, 0.3 M NaCl, 10 % Glycerol, 1 mM PMSF, 2 mM benzamidine hydrochloride, pH 7.5) and sonicated followed by centrifugation at 12,500 rpm for 30 min. We also constructed a mutant of gp560, gp560H454A that disrupts the HExxH motif essential for Zn binding, by site-directed mutagenesis using appropriate internal primers ($R519_{CAT-GCT}$: 5'-CATATTAGCTGCTGAAATTATGCAC-3' and R519GTA-CGA: 5'GTGCATAATTTCAGCAGCTAATATG 3'). Both wild type gp560 and gp560H454A were purified by successive affinity and size exclusion chromatography with buffer (50 mM tris, 0.3 M NaCl, 5 % glycerol, pH 7.5). Fractions of eluted proteins were loaded on 12 % SDS-PAGE gel (Fig. S5C).

To assess whether the purified recombinant gp560 protein is functionally active, we performed the zymography assay using 0.5 % gelatin (Himedia, RM019) as a substrate. After staining, we found a clear band on zymogram. To show effect of inhibitors, purified gp560 protein was pre-incubated separately with inhibitors (PMSF, 1,10 phenanthroline, EDTA, EGTA) at 25 °C for 20 min at 10 mM final concentration. The intensity was reduced when gp560 was incubated with the inhibitors EDTA and 1, 10 phenanthroline as expected. The proteolytic activity of gp560 was found to be concentration dependent (Fig. S5D-(i)). Also, in the presence of phenylmethylsulfonyl fluoride (PMSF), a well-known protease inhibitor, no band was observed suggesting that the observed proteolytic activity is from the purified protein. The gp560H454A mutant showed reduced activity as expected. Further to test whether Zn^{2+} is required for the activity of gp560, metal-free gp560 protein was incubated with 100 µM $ZnCl_2$ in developing buffer (100 mM Tris-HCl, pH 7.6) at 37 °C for 18-20 hours. Indeed, Zn^{2+} was able to restore the activity of wild type gp560 protein treated with EDTA and 1, 10 phenanthroline, but could not restore the activity of gp560 treated with PMSF (Fig. S5D-(ii)). This data indicates that gp560 is a potential metalloprotease which requires Zn^{2+} for its activity.

Since the purified recombinant gp560 protein is proteolytically active, we tested it for its ability to cleave the commercially available porcine brain tubulin (cytoskeleton, T238P) and also depolymerize the commercially available polymerized form of G-actin (sigma, A2522). For assessing the ability of gp560 in the depolymerization of actin, commercially available G-actin was polymerized into F-actin. For this, G-actin (2 µg) was incubated in 10x F-actin buffer (0.5 M KCl, 20 mM $MgCl_2$, 10 mM ATP, 10 mM EGTA) at RT for 60 min and then incubated with 2.7 µM concentration of the purified gp560 protein along with 0.1 µM $ZnCl_2$ at 32 °C for 6 h. After centrifugation at 35,000 *g* for 1.5 h, the supernatant (G-actin) and pellet (F-actin) fractions

were loaded on 12 % SDS-PAGE gel. As a control, polymerized F-actin alone was used. Purified recombinant gp560 protein did not cause a reduction in the amount of actin in the pellet fraction (Fig. S5E).

For assessing the activity of gp560 against tubulin cleavage or reduction of actin, purified recombinant gp560 protein was incubated with tubulin/actin in PIPES buffer (10 mM PIPES, pH 6.8, 0.1 mM GTP, 0.5 mM $MgCl_2$, 1mM $CaCl_2$, 0.1 µM $ZnCl_2$) or in G-actin buffer (5 mM tris-HCl, pH 8, 0.2 mM ATP, 0.2 mM $CaCl_2$, 0.1 µM $ZnCl_2$), respectively, for indicated time at 32 °C. Samples were separated by 12 % SDS-PAGE gel and blotted with anti-tubulin and anti-actin antibodies. Lysate collected from Mimivirus infected cells (12 hpi) and tubulin/actin alone were used as controls. The cleavage of tubulin or reduction of actin was not observed even when 2.7 µM concentration of gp560 was used for longer incubation periods of up to 2 h (Fig. S5F and S2G) suggesting that gp560 does not cleave the commercially available tubulin from Porcine brain.

References

1. Liu Y, Zhu W, Tan Y, Nakayasu ES, Staiger CJ, Luo ZQ. 2017. A Legionella Effector Disrupts Host Cytoskeletal Structure by Cleaving Actin. PLoS Pathog 13.
2. Froeliger EH, Oetjen J, Bond JP, Fives-Taylor P. 1999. Streptococcus parasanguis pepO Encodes an Endopeptidase with Structure and Activity Similar to Those of Enzymes That Modulate Peptide Receptor Signaling in Eukaryotic Cells. Infect Immun 67:5206–5214.
3. Chang PC, Kuo T-C, Tsugita A, Lee Y-HW. 1990. Extracellular metalloprotease gene of Streptomyces cacaoi: structure, nucleotide sequence and characterization of the cloned gene product. Gene 88:87–95.
4. Bianchetti L, Oudet C, Poch O. 2002. M13 endopeptidases: New conserved motifs correlated with structure, and simultaneous phylogenetic occurrence of PHEX and the bony fish. Proteins Struct Funct Genet 47:481–488.
5. Nalivaeva NN, Zhuravin IA, Turner AJ. 2020. Neprilysin expression and functions in development, ageing and disease. Mech Ageing Dev 192.
6. Turner AJ, Isaac RE, Coates D. 2001. The neprilysin (NEP) family of zinc metalloendopeptidases: Genomics and function. BioEssays 23:261–269.

Movie S1. Change in host cell morphology during Mimivirus infection. *Acanthamoeba* cells were infected with SYBR Green I stained Mimivirus at MOI 10 and video were recorded for 16 h to cover changes in host cell morphology during infection.

Movie S2. Change in host cell morphology during Marseillevirus infection. *Acanthamoeba* cells were infected with SYBR Green I stained Marseillevirus at MOI 10 and video were recorded for 24 h to cover changes in host cell morphology during infection.

Movie S3. Immediate lysis of Mimivirus-infected cells. Lysis of Mimivirus-infected cells were recorded between 16-24 hpi. In most of the cells new progenies were released through bursting.

Movie S4. Gradual lysis of Mimivirus-infected cells. Lysis of Mimivirus-infected cells were recorded between 16-24 hpi. Rarely, new progenies were released slowly without bursting of cell.

Movie S5. Lysis of Marseillevirus-infected cells. Lysis of Marseillevirus-infected cells were recorded between 24-44 hpi. Viruses were released in the form of vesicles.
